# Trees in Towns

**Published:** 1884-03

**Authors:** 


### Trees in Towns.

The question of the utility of trees in towns and cities was debated at the International Hygienic Congress held at Geneva last autumn, and has since been discussed with considerable spirit in the press of the city on the Rhone. The negative side has been supported with ability by Professor Piachaud, who favors the conclusions of the congress; Professor Goret champions the affirmative side, and has, we think, much the best of the argument. Trees are useful in centres of population, not only in affording shade from the fierce heat of the sun, but in distributing moisture in dry weather through evaporation from their foliage, and in facilitating the drainage of the soil by means of their roots. The part played by trees in maintaining the equilibrium of the atmospheric gases is also of immense importance in crowded localities.

